# DNA Repair in Haploid Context

**DOI:** 10.3390/ijms222212418

**Published:** 2021-11-17

**Authors:** Loïs Mourrain, Guylain Boissonneault

**Affiliations:** Department of Biochemistry and Functional Genomics, Université de Sherbrooke, Sherbrooke, QC J1K 2R1, Canada; lois.mourrain@usherbrooke.ca

**Keywords:** DNA repair, haploid, diploid, gametes, *Saccharomyces cerevisiae*, *Schizosaccharomyces pombe*

## Abstract

DNA repair is a well-covered topic as alteration of genetic integrity underlies many pathological conditions and important transgenerational consequences. Surprisingly, the ploidy status is rarely considered although the presence of homologous chromosomes dramatically impacts the repair capacities of cells. This is especially important for the haploid gametes as they must transfer genetic information to the offspring. An understanding of the different mechanisms monitoring genetic integrity in this context is, therefore, essential as differences in repair pathways exist that differentiate the gamete’s role in transgenerational inheritance. Hence, the oocyte must have the most reliable repair capacity while sperm, produced in large numbers and from many differentiation steps, are expected to carry de novo variations. This review describes the main DNA repair pathways with a special emphasis on ploidy. Differences between *Saccharomyces cerevisiae* and *Schizosaccharomyces pombe* are especially useful to this aim as they can maintain a diploid and haploid life cycle respectively.

## 1. Introduction

Maintenance of genome integrity is a permanent cell challenge as both intra- and extracellular conditions can lead to chemical alterations of nucleotides or their sequence. Proper repair mechanisms have evolved so as to maintain a balance between maintenance of cellular function and adaptative processes improving fitness. For obvious reasons, germline cells must be especially proficient at this task as diversity must be transmitted while maintaining the gametes’ integrity through the many differentiation steps. In addition, anisogamy, whereby the two sexes produced highly different and specialized haploid gametes, enhances diversity. Indeed, while diploidy allows gene versions compensation and error-free repair, haploidy is expected to create more variation as there is no templated repair for homologous recombination. Ploidy is therefore a key element to consider for genome plasticity or stability. The following review discusses the DNA repair and genome maintenance processes linked to the ploidy state by comparing two unicellular eukaryotes models, yeasts *Saccharomyces cerevisiae* (*S. cerevisiae*) and *Schizosaccharomyces pombe* (*S. pombe*), while considering the case of the mammalian gametes.

## 2. General Consideration Regarding Ploidy States

Since the turn of the century, knowledge regarding the advantages of different ploidy states has been increasing thanks to the study of lower eukaryotes. Thus, fission and budding yeasts have proved to be valuable models because they, respectively, represent the impact of haploid and diploid genomes on evolution and genetic integrity. The ploidy choice for lifespan appears to be strongly related to the evolution and maintenance of genetic integrity [1,2,3,4]. It is well known that diploidy confers greater fidelity to replication of both genomic and mitochondrial DNA, in particular, to attenuate single-nucleotide mutations. However, large chromosomal rearrangements or deletions occur more frequently in the diploid budding yeast impacting cellular fitness. Although there is twice the amount of genetic material in diploid cells, therefore statistically twice as many possible mutations [5,6], observations show that there are only 1.4 times more mutations in diploid cells than in haploid cells [7]. Such a bias is perhaps linked to the faster replication in the haploid state allowing a much shorter repair time. Errors mainly involve AT or CG transversions in late replicating genes [8]. In addition to this mutation bias, acquisition of a recessive mutation that would clearly alter the cell survival or mutation load is reduced by diploidy because two such similar recessive mutations must happen over both alleles to alter the phenotype. However, such recessive mutations will be eliminated in the population, a process coined “heterozygote advantage”. 

On the other hand, this would prevent diploid cells from positively adapting to a changing environment [9]. Diploid cells can nevertheless accumulate heterozygous mutations, creating a pool of sequence variants that can be useful in reaction to a changing environment, whereas haploid cells either die or survive. Larger chromosome rearrangements in the diploid cells should be explained by the homologous recombination between chromosomes, which represents the main difference from haploid cells. However, the deletion of RDH54, a protein necessary for recombination between homologous chromosomes, does not alter the mutation rate of diploid cells [7]. This observation may be reconciled when considering the fission yeast *S. Pombe*, which maintains a haploid life cycle. *S. pombe* seems to be protected against the instability of the haploid life cycle by spending most of its time in the G2 phase. Indeed, in the G2 phase, sister chromatids are present allowing error-free repair of Double Strand Break (DSB), Homologous Recombination (HR). Although the alternative to HR is the error-prone Non-Homologous End-Joining (NHEJ). *S. pombe* promotes HR through stalling at the G2 phase. Deletion of NHEJ repair protein (PKu70*^Sp^* or Lig4*^Sp^*) displays low sensitivity to DSB-inducing genotoxins, indicating that even outside the G2 phases, *S. pombe* may not rely on NHEJ for damage repair. However, these observations are in contrast to the higher mutation rate observed in haploid cells compared to diploids. For example, DNA microsatellites are 100 times more stable in diploid cells [10]. Another example is the higher rate of mutation found in mitochondrial DNA of haploid cells [7]. 

One potential explanation is the higher number of mitochondria in diploid cells while mitochondria often mate, mixing alleles versions, rapidly reducing heteroplasmia. In addition, polyploid cells evolve faster than haploids, in relation to chromosomal rearrangement and aberrant condition of polyploid cells [11]. It, therefore, appears that the difference in the amount of DNA between the ploidy states only partially explains the difference in the mutation rate. Although haploidy allows a faster evolution, this condition is protected by some elements preventing excessive genome instability. Similarly, anisogamy in mammals results in very numerous haploids spermatozoa versus a single diploid ovum before fertilization. As stated above, genetic stability of the ovum is provided by diploidy and a complete array of repair systems, while the haploid character of sperm and the condensed chromatin may provide the proper context for DNA repair errors at the origin of the well-known male bias in the transmission of de novo mutations. In that sense, the ovum stands as the guardian of heredity while spermatozoa are providers of diversity.

## 3. DNA Repair Systems Comparison between *S. cerevisiae* and *Sc. pombe*

### 3.1. Nucleotide Excision Repair

#### 3.1.1. General Mechanism

**Figure 1:**
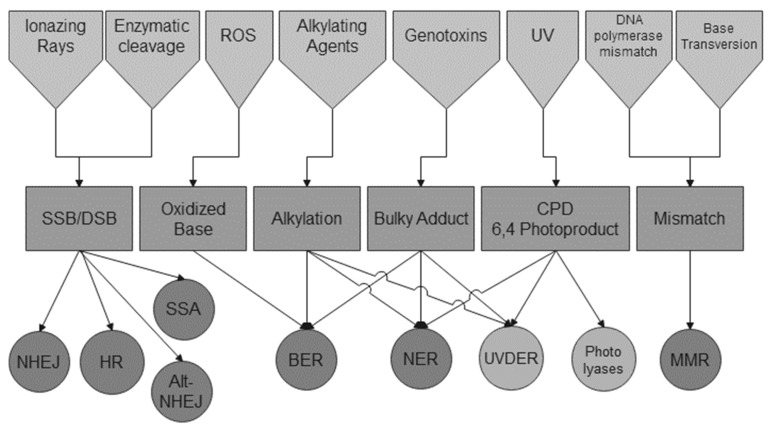
Origin, type, and repair pathways of DNA damages. Various inducers of DNA damage are represented (top line), whereas the type of damage is represented for each (middle line). The circles (bottom line) represent known repair pathways. Darker circles represent pathways found through the eukaryotic domain whereas circles in light grey are those specific to yeast. (Abbreviations: ROS: Reactive Oxygen Species; SSB: Single Strand break; DSB: Double-Strand Break; CPD: Cyclo-Pyrimidine Dimer; NHEJ: Non-Homologous End-Joining; HR: Homologous Recombination; SSA: Single Strand Annealing; Alt-NHEJ: Alternative NHEJ; BER: Base Excision Repair; NER: Nucleotide Excision Repair; UVDER: UV Damage Excision Repair; MMR: Mismatch Repair).

**Figure 2:**
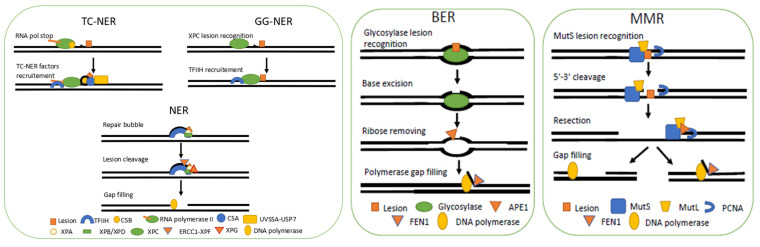
DNA base repair pathways. Simplified schematic representation of Nucleotide Excision Repair (NER), Base Excision Repair (BER), and Mismatch Repair (MMR). Transcription Coupled NER (TC-NER) starts with RNA pol II stalling in front of a lesion. Then CSB recruits CSA and UVSSA-USP7 allowing the TFIIH docking. Global Genome NER (GG-NER) is initiated by XPC lesion recognition. Through a cascade of events, TFIIH is recruited. Both NER pathways converge when TFIIH is recruited. Thanks to its helicases, XPB/XPD, and XPA, TFIIH creates a DNA repair bubble exposing the lesion. Then ERCC1-XPF and XPG cleave DNA around the lesion. The gap is then filled by DNA polymerase. In BER, glycosylase targets the lesion and cleaves base-deoxyribose glycosidic bounds. When Glycosylase is released from DNA, APE1 can now bind to cleave the faulty deoxyribose. DNA polymerase fills the gap and displaces some of the damaged strands. The resulting 3′ tail is then removed by FEN1. In MMR the mismatch is targeted by MutS, then recruiting MutL. Then an endonuclease cleaves DNA, and a resection occurs. Finally, polymerase fills the gap and could create a 3′ overhang tail.

Nucleotide excision repair (NER) resolves various substrates resulting in structural alterations of DNA helix (Figure 1) [12]. NER can be divided into two sub-pathways: transcription-coupled NER (TC-NER) and global-genome NER (GG-NER). Although GG-NER uses self-assessment of DNA damage, TC-NER uses hindrance of the RNA polymerase transit to detect bulky adducts (Figure 2). This transcription-coupled mechanism ensures the elimination of mutations during transcription by the so-called transcriptional mutagenesis. Whereas TC-NER is dedicated to transcribed strands of active genes, GG-NER detects DNA helix disturbing damages in both transcribed and non-transcribed strands [13].

TC-NER is triggered by RNA polymerase blockage. RNA polymerases are exceptionally processive, and cover between 50 up to 90% of the genome depending on the cell’s transcriptional state, providing a reliable scanning process for damage. In addition to bulky adducts, pol II arrest can result from a wide variety of potential hindrances (Figure 1) including nicks, gaps, abasic sites, collisions with the replication complex, stable DNA-RNA dimers, or non-B DNA [14]. CSB*^Hs^* (Cockayne Syndrome B protein) is the first NER factor to be recruited. When the pol II stops, the CSB*^Hs^*-pol II interaction becomes stronger and CSB*^Hs^* wraps DNA around itself hence decreasing pol II-DNA interaction [15]. Then CSB*^Hs^* recruits CSA*^Hs^* (Cockayne Syndrome A protein) [16] at the lesion site allowing the docking of UVSSA-USP7*^Hs^*. The latter is a ubiquitin ligase complex that activates CSA*^Hs^*, in contrast to CSB*^Hs^* which is ubiquitinated and targeted for degradation [17]. Following repair, USP7*^Hs^* removes ubiquitination of all NER components so they can be recycled. The second step in TC-NER is done by the transcription factor TFIIH*^Hs^*, which binds DNA downstream of pol II*^Hs^* and provides an “assessment” of the lesion [18].

GG-NER targets larger lesions, typically more than one nucleotide in size, as this dramatically changes the DNA helix thermodynamics [19]. XPC-Rad23b*^Hs^* (XPC*^Hs^* complex, Xeroderma Pigmentum C protein) is the first complex to bind the DNA lesion. The XPC*^Hs^* complex may detect DNA lesions as they create a single strand local transition [20]. Other than the scanning mechanism, alternative recognition mechanisms by XPC*^Hs^* do exist [21,22,23,24]. XPC*^Hs^* SUMOylation, and re-ubiquitinoylation, are then required for GG-NER progression [25,26]. Once activated, XPC*^Hs^* complex recruits TFIIH*^Hs^*. TFIIH*^Hs^* can help XPC*^Hs^* to discriminate between lesion types since XPC*^Hs^* is easily misled by a base mismatch [27].

TC-NER and GG-NER repair pathways converge once TFIIH*^Hs^* is recruited. TFIIH*^Hs^* core subunits XPB*^Hs^* and XPD*^Hs^* create a “repair bubble” of 20-30 nucleotides surrounding the lesion [28]. A second proofreading process involves XPA*^Hs^*. XPA*^Hs^* normally stimulates the helicase activity of TFIIH*^Hs^* but can decrease this activity in the presence of a lesion. XPA*^Hs^* recruits RECCI-XPF*^Hs^* [29] a nuclease that will cleave 5′ of the lesion [30]. From its interaction with the TFIIH*^Hs^* complex, XPG*^Hs^* endonuclease can then cleaves 3′ of the lesion [31,32] and replaces XPC*^Hs^*. XPG*^Hs^* plays a structural role in complex with TFIIH*^Hs^* but acquires endonuclease activity once ERCC1-XPF*^Hs^* is recruited by XPA*^Hs^* 5′ to the lesion. ERCC1-XPF*^Hs^* is the first to nick DNA followed by XPG*^Hs^* [33]. After removal of the damaged nucleotide, DNA polymerases can fill the gap, while ligase seals the nick.

#### 3.1.2. NER in Yeast

As for mammals, NER in yeasts is divided into two sub-pathways (Figure 2). However, *Sc. pombe* NER is less documented as redundancy with UVDER (UV Damage Excision Repair, see below) leads to phenotypic confusion. In *S. cerevisiae,* TC-NER is triggered after RNA polymerase stops, activating either Rad26*^Sc^* (CSB*^Hs^* homolog) or Rpd9*^Sc^*, an RNA pol II*^Sc^* subunit-specific of budding yeast [34]. Rad26*^Sc^* deletion decreases mRNA synthesis, in agreement with its transcription promoting activity over direct damage repair (8-oxoG, 3-MeA, abasic site) [35]. This may, however, lead to “transcriptional mutagenesis”. If the lesion creates a major hindrance, Rad26*^Sc^* then initiates chromatin remodelling and the recruitment of NER partners. An alternative TC-NER mechanism involves RNA pol II subunit Rpb9*^Sc^*, which can also recruit the NER machinery. In *Sc. pombe*, Rhp26*^Sp^* (CSB*^Hs^* homolog) deletion displays an important increase in UV sensitivity [36], demonstrating the prominent role of the TC-NER pathway in UV resistance. In *S. cerevisiae*, GG-NER starts, with the Rad23-Rad33*^Sc^* (Rad23b*^Hs^* homologs) complex that can transit over DNA until a UV lesion is encountered. Rad4*^Sc^* (XPC*^Hs^* homolog) is then recruited to initiate NER [37,38]. While TFIIH*^Sc^* is already positioned at the lesion in TC-NER, a control step is needed in GG-NER. This control step uses RPA*^Sc^* (Replication protein A), which binds intact DNA, and Rad14*^Sc^* (XPA*^Hs^* homolog) which binds damaged DNA [39]. Without such a control step, NER will abort before the final dual incision [40]. GG-NER in *Sc. pombe* uses two XPC*^Hs^* homologs: Rhp41*^Sp^* and Rhp42*^Sp^* [41,42]. Whereas Rhp41*^Sp^* has a major role in Cyclo-Pyrimidine Dimers (CPDs) removal in both transcribed and non-transcribed genes, Rhp42*^Sp^* is only active over non-transcribed genes [41,42,43]. Following damage detection by either GG-NER or TC-NER, Rad2*^Sc^*/Rad13*^Sp^* (XPG*^Hs^* homologs) then cleaves 3′ of the lesion, whereas Rad1*^Sc^*-Rad10*^Sc^*/Rhp16*^Sp^*-Swi10*^Sp^* (ERCC1-XPF*^Hs^* homologs) cleave in 5′, respectively in *S. cerevisiae* and in *Sc. pombe* [44,45,46].

### 3.2. Base Excision Repair

#### 3.2.1. General Mechanism

Base excision repair (BER) is the DNA damage repair pathway in charge of resolving chromatin lesions not associated with structural alterations of the DNA helix [47]. BER mainly recognizes oxidized base (8-oxoG), deaminated base (uracil), and alkylated base (Figure 1). BER also takes charge of the abasic sites. Apuric or apyrimidic sites (AP) are indeed the most frequent lesions encountered in a stationary state [48,49]. BER pathway uses glycosylases which target abnormal bases and cleave β glycosidic bonds between riboses and bases. Glycosylases are generally evolutionary conserved, from bacteria to higher eukaryotes [50]. In mitochondria, BER also exists and uses different isoforms, resulting in the most robust DNA repair in mitochondria [51,52].

Glycosylases are generally small proteins harbouring a unique catalytic domain with high substrate specificity. Although most of these enzymes have unique glycosylase activity (monofunctional), some also have β lyase, or β/Δ lyase activities able to cleave in 3′ or 5′P, or both (bi- trifunctional) [51,52]. Glycosylases first bind the minor groove, inducing a kink in DNA, then flip the faulty base out of the helix and separate the base from the ribose [53]. Irrespective of their host organism, glycosylases are fast to catalyse the glycosidic bond cleavage, but then disengage from DNA with much slower kinetics [54,55]. This dual kinetic mode may therefore hide AP sites, which can lead to apoptosis when reaching a given threshold. If the glycosylase is monofunctional, AP site-specific endonuclease will assist in cleaving 3′ of the deoxyribose, and DNA pol will remove the 5′ deoxyribose phosphate (dRP) during DNA neosynthesis [51,56,57]. On the other hand, if the glycosylase also possesses a lyase activity, this will result in DNA nicking 5′ to the damaged deoxyribose. The 3′ dRP is then processed by AP endonuclease, leaving a functional 3′OH, the initiation site for DNA neosynthesis, and a 5′-diphosphate (5′-PP) thereby facilitating DNA pol binding (Figure 2) [58]. BER comprises two sub pathways: short and long patch repair. Either short or long patch repair pathways are used by the cell depending on the lesion size or the glycosylase involved. One single nucleotide is excised in short patch repair whereas long patch BER involves two or more nucleotides [59]. The main BER pathway is usually the short patch as it does not require the replication machinery typical of long patch repair. The short patch process uses glycosylase, the AP endonuclease, DNA polymerase, and DNA ligase. Long patch repair is mainly used during replication and uses a glycosylase and the AP endonuclease, but usually relies on DNA polymerase, PCNA (Proliferating Cell Nuclear Antigen), flap endonuclease from the replication machinery, as well as DNA ligase [51]. In a long patch repair pathway, the basic catalytic pocket of DNA polymerase can contain some ssDNA while the repair synthesis bypasses the lesion site creating a 5′ overhang tail with the old DNA. The Flap endonuclease (mammalian FEN1*^Hs^*, *S. cerevisiae* Rad27*^Sc^*, *Sc. pombe* Rad2*^Sp^*), which possesses a 5′flap endonuclease activity and 5′ to 3′ exonuclease activity, will then remove this 5′ tail [60].

#### 3.2.2. BER in Yeast

Six DNA glycosylases have been characterized in *S. cerevisiae*: monofunctionals, Ung1*^Sc^* and Mag1*^Sc^*, Mag2*^Sc^*; and bifunctionals, Ngt1*^Sc^*, Ngt2*^Sc^*, and Ogg1*^Sc^*. In *Sc. Pombe*, monofunctionals, Ung1*^Sp^*, Thp1*^Sp^*, Mag1*^Sp^*, Mag2*^Sp^*, and Myh1*^Sp^*, and the bifunctional, Nth1*^Sp^* have been characterized (Table 1). Ung1*^Sc^*, Ung1*^Sp^*, and Thp1*^Sp^* are Uracil DNA Glycosylases (UDG) recognising uracil resulting from cytosine deamination [61]. Thp1*^Sp^* also recognizes T:G or U:G mismatches, but is also known to process 5-fluorouracil, 3,N4-ethanocytosine and 5-hydroxyuracil [62]. In addition, Thp1*^Sp^* recognizes deaminated purines (xanthine and oxanine from guanine, hypoxanthine from adenine) [63]. *S. cerevisiae’s* Mag1*^Sc^* and Mag2*^Sc^* are expressed after exposure to alkylating agents [64]. Mag1*^Sc^* is also able to cleave normal bases, usually guanine [65]. In *Sc. Pombe,* Mag1*^Sp^* and Mag2*^Sp^* share 44,8% identity. Although Mag1*^Sp^* initiates the alkylation repair, Mag2*^Sp^* may also plays a role in the process [66]. Unlike *S. cerevisiae* Mag1*^Sc^*, deletion of Mag1*^Sp^* only slightly increases alkylating agents’ sensitivity [67]. However, Rad13*^Sp^*, Rad16*^Sp^* or Rhp51*^Sp^* deletion mutants are hypersensitive to alkylation, suggesting an important contribution of NER or HR to this type of damage in *Sc. pombe* [67,68]. Although, mutants of both *Sc. pombe* BER endonucleases, Apn2*^Sp^* or Nth1*^Sp^*, are hypersensitive to alkylation, confirming BER in alkylation repair [69,70,71]. Surprisingly, Mag1*^Sp^* deletion leads to a 3-fold increase in intrachromosomic recombinations [68], suggesting that HR is somehow involved in alkylation repair in *Sc. pombe*. In *S. cerevisiae* Ntg1*^Sc^* is a bifunctional glycosylase located in the mitochondrion whereas Ntg2*^Sc^* is nucleolar. However, only Ntg1*^Sc^* is upregulated during oxidative stress [72]. These enzymes can process a wide variety of the oxidized pyrimidine and purine lesions [73]. However, Ntg1*^Sc^* is the sole enzyme able to resolve 8-oxoG whereas Ogg1*^Sc^* processes only Fapy-G and 7,8 dihydro-8-oxoG [74]. Surprisingly, an Ogg1*^Sc^* homolog is not found in *Sc. Pombe* [75]. Instead, Nth1*^Sp^* is in charge of resolving 8-oxoG in a mismatch situation. Nth1*^Sp^* seems to process a wide variety of substrates including 8-hydroxycytosine, thimidineglycol, and 8-hydroxyuracil [76]. 

Due to its bifunctional activity, Nth1*^Sp^* acts upstream of the Apn2*^Sp^* AP endonuclease, as it leaves a 3′ protected end, which is removed by the phosphoesterase activity of Apn2*^Sp^* [69]. In fission yeast, Nth1*^Sp^* also resolves AP sites resulting from the activity of both Mag1*^Sp^* and Mag2*^Sp^*, therefore playing a central role in BER [71]. However, in contrast to mammals NTHL1*^Hs^*, or *S. cerevisiae* Ntg1*^Sc^*, Nth1*^Sp^* is seemingly absent from mitochondrion. Finally, Myh1*^Sp^*, is a glycosylase specific to *Sc. pombe* and in charge of mismatches found at adenines (A:G, A:8-oxoG, 2-aminopurine:G, 2-aminopurine:A) [77]. Myh1*^Sp^* may also process G:8-oxoG, preventing C:G to G:C transversion [78]. A prominent role for Myh1*^Sp^* is binding to PCNA*^Sp^* and the PCNA-like heterotrimer Rad9*^Sp^*/Rad10*^Sp^*/Hus1*^Sp^* [79,80], which are involved in DNA lesion checkpoint. After base eviction, the AP site is processed by the two AP endonucleases found in yeasts. Apn1*^Sc^* and Apn2*^Sc^* are the two AP endonucleases found in *S. cerevisiae* although Apn1*^Sc^* is responsible for >95% of the total in vivo activity [81]. Apn1*^Sc^* cleaves 5′ to the lesion and possess a 3′ phospho-esterase activity able to resolve 3′dRP, 3′ phospho-glycolate (3′PGA) or 3′P 3′ dirty ends. Both *S. cerevisiae* AP endonucleases are 3′ tyrosyl DNA phospho-esterase with a weak 3′ exonuclease activity so they can resolve Topo1*^Sc^*-DNA adducts [82]. In *Sc. pombe*, Apn2*^Sp^* is the major AP endonuclease. Unlike Apn2*^Sp^*, Apn1*^Sp^* does not need specific subdomains to process PCNA*^Sp^* or Top3*^Sp^* catalytic hindrance, Apn1*^Sp^* may represent a proper backup to Apn2*^Sp^* at 3′ blocked end, but to a limited extent as Apn1*^Sp^* is present in both nucleus and cytoplasm, unlike Apn2*^Sp^* [83]. Uve1*^Sp^*, an UVDER enzyme, may also be involved in the processing of AP sites [84], especially as it is present in both the nucleus and mitochondrion [85].

### 3.3. Mismatch Repair

#### 3.3.1. General Mechanism

DNA polymerases can induce from 10^−5^ to ~10^−4^ mismatch per base given their own exonucleolytic proof-reading activity. The mismatch repair (MMR) system increases repair fidelity by about ~100–1000 times [86]. The MMR pathway is in charge of mismatches but also resolves intra-strand loops. The MMR pathway was first described in *Escherichia coli* (*E. coli)* which represented a key model to decipher the sequential events of MMR: MutS first detects the mismatch, MutL confirms the helix deformation induced by the mismatch and MutH endonuclease cleaves the faulty base. DNA resection then occurs, followed by DNA polymerase gap filing and nick sealing by DNA ligase (Figure 2). While eukaryotic MutS and MutL exist as heterodimers [87,88], the MMR-specific MutH endonuclease is seemingly absent in eukaryotes and some bacteria. Lesion recognition by MMR is achieved by two heterodimeric homologs: MutSα and MutSβ. Whereas MutSα is in charge of small mismatches and helix extrusions of about 1–4 nt in size, MutSβ exclusively recognizes larger extrusions of 2–10 nt [89]. Loss of MutSβ can easily be compensated for by MutSα. Lesion recognition by MutSα is indeed prominent in the MMR pathway [90]. Once the MutS complex becomes bound to DNA, it recruits MutLα or MutLβ. During recombination, MMR proteins act to repair mismatch in DNA heteroduplexes, remove 3′ non-complementary tails produced during resection, and prevent recombination between divergent sequences. MutLγ and MutLβ have been shown to play a role in meiotic HR [91]. MutLγ is mainly used during meiotic HR, where MutSγ activates it. In the mismatch repair, the endonucleolytic cleavage is done over the weakest strand, admitting the one containing a mismatch, in both 3′ and 5′ flanking the lesion. The mechanism directing strand selection is still unknown but may involve PCNA, which is still bound to the neosynthetised strand or may result from hypomethylation of the new strand [92]. Although a eukaryotic MutH has yet to be identified, the human PMS2*^Hs^*, part of MutLα*^Hs^*, have been shown to possess a latent endonuclease activity. Removing its endonuclease motif abolishes the mitotic function of MutLα*^Hs^* [93]. After incision, a fragment of DNA is excised by Exo1*^Hs^*. Resection by Exo1*^Hs^* is greatly enhanced by association with MutSα*^Hs^* and ATP [94], as it removes only a few nucleotides when acting independently. After resection, DNA polymerase fills in the gap while DNA ligase ligates the 3′ end to complete the process. An alternative pathway to MMR involved DNA polymerase, whereby the faulty strand is displaced during DNA synthesis [95].

#### 3.3.2. MMR in Yeast

In *S. cerevisiae*, while MutSα*^Sc^* targets mismatches, MutSβ*^Sc^* is mainly in charge of long insertion-deletion loops (IDLs), although redundancy exists between both as MutSβ*^Sc^* may substitute for defective MutSα*^Sc^* but to a limited extent [96]. MutSα*^Sc^* initiates repair of all mismatches except C:C [97]. IDLs are repaired more efficiently than mismatches as MutS functional redundancy facilitates the repair of small IDLs [98,99]. *Sc. pombe* possesses four MutS homologs (Msh1*^Sp^*, Msh2*^Sp^*, Swi4*^Sp^*, Msh6*^Sp^*) [100]. MutSα*^Sp^* (Msh2*^Sp^*-Msh6*^Sp^*) is sufficient to support *Sc. pombe* MMR, regardless of the lesion type. However, Swi4*^Sp^*, likely acting in complex with Msh2*^Sp^*, is involved in mating-type switching and some recombination processes, as demonstrated for MutSβ*^Sc^* in *S. cerevisiae* [101,102]. Nevertheless, although *Sc. pombe* MutSβ*^Sp^* is solely implicated in recombination, the MMR function, typical of the *S. cerevisiae* MutSβ*^Sc^*, has apparently been lost. The MutSγ*^Sc^* complex in *S. cerevisiae* is only expressed during meiosis and is linked to recombination [103,104]. In yeast, MutLγ*^Sc^* is the main Holliday Junctions resolvase [105], symmetrically nicking Holiday Junctions. MutL homologs in budding yeast includes MutLα*^Sc^* (Mlh1*^Sc^*-Pms1*^Sc^*), MutLβ*^Sc^* (Mlh1*^Sc^*-Mlh2*^Sc^*) and MutLγ*^Sc^* (Mlh1*^Sc^*-Mlh3*^Sc^*) [106]. While MutLα*^Sc^* interacts with MutSα*^Sc^* and MutSβ*^Sc^* to process most of MMR, MutLγ*^Sc^* only interacts with MutSγ*^Sc^* to resolve meiotic crossing-over. *Sc. pombe* contains only one MutL homolog (Mlh1*^Sp^*-Pms1*^Sp^*) [100]. Thus, MutSα*^Sp^* and MutLα*^Sp^* should be sufficient for a functional MMR machinery in fission yeast, as it seems to be the case in *Drosophila* and *Caenorhabditis*. In *S. cerevisiae,* Exo1*^Sc^* mutants were found to be slightly mutagenic, owing to its “error-free damage bypass” function, as described by Tan et al. [107]. Considering the weak impact of Exo1*^Sc^* deletion, one may surmise that other nucleases may be involved such as Rad27*^Sc^* or from the proofreading activity of DNA polymerases Δ or ε*^Sc^* [107,108]. In contrast, Exo1*^Sp^* deletion in *Sc. pombe* generates a strong mutator phenotype, probably as a result of its role in GT repeat maintenance in an MMR-independent pathway [109].

A short patch MMR pathway is described in fission yeast and involves the NER machinery [110]. Indeed, Rhp14*^Sp^*, Swi10*^Sp^*, Rad13*^Sp^*, Rad15*^Sp^*, and Rad16*^Sp^* are important NER effectors components in this short patch repair pathway [111,112,113]. The NER machinery may therefore play a broader role and supports MMR pathways in specific cases. The first clear evidence is the NER requirement for C:C mismatches, which cannot be repaired by MMR [111,113]. Hence, in the absence of MMR, C:C mismatches are quickly repaired, so NER seemingly processes such damage with high efficiency. Interestingly, the NER Rhp14*^Sp^* binds to various mismatches with similar affinity to C:C mismatches [112]. Defective NER has also been shown to induce a slight increase in reversion of GT repeats, suggesting a role for NER in *Sc. pombe* microsatellites maintenance [112]. Short patch MMR exists in *S. cerevisiae* but is seemingly independent of NER [114]. In *S. cerevisiae*, NER mutants show an increase in reversion rate but are mainly associated with flanking nucleotides substitutions, suggesting a more complex mechanism, likely originating from a bypass by the error-prone DNA pol ζ [115]. In vitro evidence indicated that NER may also play a role in mismatch repair in other species including *drosophila* [116,117], *E. coli,* and humans [118].

### 3.4. Double Strand Breaks (DSBs) Repair Pathways Selection

#### 3.4.1. DSB Regulation in Mammals

In humans, about 1 double-strand break (DSB) arises in 10^8^ base pairs, so around 10–30 DSBs are generated per cell, per cell cycle, resulting from endogenous or exogenous factors [119,120]. The two main DSBs repair pathways are Homologous Recombination (HR) and Non-Homologous End-Joining (NHEJ). These pathways are quite contrasted in terms of quality and timing of repair during the cell cycle. HR relies on sister chromatids to repair DNA, providing an almost error-free mechanism. HR is therefore dependent on the cell cycle, being most active during S-G2 phases. In contrast, NHEJ is an error-prone system, inducing sequence variations, and is mainly active during G1-G2 phases, although its components are found during the whole cell cycle [121,122]. Whereas HR needs hours to complete, NHEJ can repair DSBs within half an hour given the high processivity of its enzymes [123]. Upon inactivation of NHEJ (mutation of Ku, Lig4, or XRCC4 proteins), HR becomes the prominent DSBs repair pathway [124]. If sequence homologies however exist at DSBs ends, other repair pathways may be used such as Single Strand Annealing (SSA), Micro-homology End-Joining (MMEJ), and an Alternative NHEJ (Alt-NHEJ) pathway. SSA requires at least a repetition of two nucleotides to be present at both ends of the DSB. However, the two-nucleotides homology can also be used by MMEJ or alt-NHEJ. MMEJ is frequently used even though HR proteins are present or in cases of defective HR [125]. A choice must be made between NHEJ, HR, SSA, MMEJ, and alt-NEHJ (Figure 2). Initially, the DSB may undergo a direct repair by NHEJ, or resection may occur. If resection is chosen, then a competition takes place between HR, SSA, MMEJ, and alt-NHEJ depending on resection size [126,127,128]. Resection is inhibited either by the NHEJ Ku*^Hs^* binding at DNA ends or by both the p53 binding protein 1 (53BP1*^Hs^*) or the Replication timing regulatory Factor 1 (RIF1*^Hs^*), which are known to be resection antagonists [129]. Many such down regulators of resection lead to the predominance of NHEJ. If a resection still occurs, a competition between other repair systems begins. The shift between HR and SSA, MMEJ, and Alt-NHEJ is dependent on the cell cycle, and particularly the cyclin-dependent kinases (CDK*^Hs^*). Indeed, during S or G2 phases, CDK*^Hs^* can phosphorylate CtIP*^Hs^* (C-terminal-binding protein-interacting-protein) thereby facilitating its interaction with BRCA1*^Hs^* to promote HR [130]. In the case of resection, it is done before the S phase and DSB repair will be achieved by SSA or MMEJ [131]. Although most of the MMEJ promoting factors also promote SSA repair [132], SSA will be preferred for smaller resection sizes. Alternative NHEJ also needs a micro-homology of 2–20 nt [133].

#### 3.4.2. DSB Regulation in Yeast

Yeast regulation of DSBs repair is relatively similar to that of mammals. However, in this case, the presence of sister chromatid is not the main factor in choosing between HR and NHEJ. Indeed, HR is regulated through the cell cycle by post-translational modifications, degradation, and re-localization of its effectors. In *S. cerevisiae,* some HR genes are even regulated by the presence of DNA damage itself [134,135]. In contrast, YKu70*^Sc^* is mainly transcribed in early G1 and almost undetectable in the S phase. In post-replicative cells, HR proteins are downregulated by Clb-CDK-inhibitors*^Sc^* or Sic1*^Sc^* overexpression [136]. A few minutes after DSB formation, YKu*^Sc^* heterodimers and MRX complexes become recruited independently [137]. MRX complexes are essential to maintain both DNA ends physically closed and protected [138], whereas YKu*^Sc^* inhibits DNA end resection [139]. As in mammals, HR-NHEJ balance is determined by resection initiation, with YKu*^Sc^* competing with MRX complex and Sae2*^Sc^* endonuclease (CtIP*^Hs^* homolog) [140]. Moreover, MRX is ambivalent regarding YKu*^Sc^*. It seems to stabilise YKu*^Sc^* on DSBs, but also destabilising it [141]. In addition, YKu*^Sc^* dissociation from unrepaired DSBs is dependent on the formation of the MRX complex, suggesting that the latter can complete the NHEJ repair process [137]. However, YKu*^Sc^* is able to prevent extensive resections in the absence of either the MRX complex or Sae2*^Sc^*, while the MRX complex is dispensable to initiate HR in the absence of YKu*^Sc^* since Exo1*^Sc^* will carry out resection [139]. In the particular case of the fission yeast, despite being a haploid organism, it spends most of its time in the G2 phase, where sister chromatids are present allowing preferential selection of HR. Thus, in contrast to mammals, HR is the major DSBs repair pathway in yeast. Unlike NHEJ mutants (PKu70*^Sp^* or lig4*^Sp^* mutants), HR mutants were shown to be much more sensitive to genotoxins [142]. However, in G1 synchronised cells, NHEJ is 7 to 10 times more important than in asynchronous cells, and PKu70*^Sp^* mutants become far more sensitive to ionising radiations [143]. This demonstrates the primary role of NHEJ in G1 phasis when sister chromatids are absent although HR remains the predominant DSBs repair pathway in yeast. However, loss of PKu70*^Sp^* increases chromosomal rearrangements from DSBs without changing the gene conversion ratio, suggesting competition between HR and NHEJ despite the lack of PKu70*^Sp^* [144]. In contrast to *S. cerevisiae*, MRX*^Sp^* mutation (deletion of Rad32*^Sp^* or Rad50*^Sp^*) does not alter NHEJ activity [145]. Moreover, Rad32-Rad50*^Sp^* double mutants are unable to proceed beyond the S-phase DNA damage checkpoint [146] showing a clear prevalence of HR. In *S. cerevisiae*, some proteins become expressed in order to promote HR. The rad55-Rad57*^Sc^* complex is also found to promote HR in *S. cerevisiae* [147]. In *S. pombe*, two Rad52*^Sc^* homologs are known: Rad22*^Sp^* and Rti1*^Sp^*. Rad22*^Sp^* is required for mating-type switching and DNA repair [148] and shares high sequence homology with Rad52*^Hs^* [143]. By itself, Rad22*^Sp^* is able to bind DSB. Rti1*^Sp^* mutant increases sensitivity to ionising radiations as much as in Rti1/Rad22*^Sp^* [149,150] double mutants. Thus, although NHEJ, SSA, MMEJ, and Alt-NHEJ do exist in yeast, HR remains the major repair mechanism regardless of the ploidy state. Details of DSB repair processes are provided in the following sections.

### 3.5. Homologous Recombination

#### 3.5.1. General Mechanism

**Figure 3:**
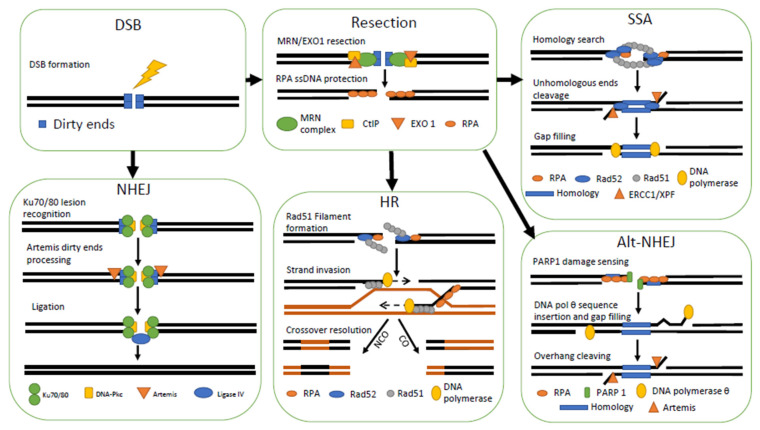
Double-Strand Breaks (DSBs) repair pathways. The repair of a DSB begins with DNA ends resection or direct ligation through Non-Homologous End-Joining (NHEJ). If resection does occur, Homologous Recombination (HR), Single Strand Annealing (SSA), and Alternative-NHEJ (Alt-NHEJ) compete to repair the DSB. NHEJ begins with Ku dimers binding to DNA ends and recruiting of DNA-Pkc. Artemis endonuclease is then recruited in order to clean dirty DNA ends, allowing the direct ligation by Ligase IV. Resection is carried out by exonuclease I (EXO1) but initiated by the MRN complex, assisted by CtIP. Single strand DNA is then protected by RPA. In HR, Rad52 will displace some RPA to allow the formation of Rad51 filament. Rad51 filament will find a homologous sequence and start strand invasion. DNA polymerase then fills gaps by using homologous chromatid as a template. Resolution of Holliday Junction leads to Chromosomal Exchange (CO) or Non-Chromosomal Exchange (NCO). SSA also uses Rad52 and Rad51, but the filaments target homologies surrounding the DSB. When hybridization occurs, unpaired 5′ overhangs are removed by ERCC1/XPF. Finally, polymerase fills the gap. Alt-NHEJ simply uses thermodynamics for annealing close to the DSB. Then DNA polymerase θ fills the gap.

The first step in HR is the formation of a single strand 3′ tail by resection from the original DSB (Figure 3). This initial step is mediated by the Mre11*^Hs^*-Rad50*^Hs^*-NBS1*^Hs^* (MRN*^Hs^*) complex and CtIP*^Hs^* in humans. The 3′ tail resulting from large 5′->3′ DNA resection is performed by Exo1*^Hs^* [151]. The main characteristic of HR is the single strand invasion over the sister chromatid. Rad51*^Hs^* is the RecA bacterial orthologue in eukaryotes with a DNA-dependent ATPase activity able to form a nucleoprotein-DNA filament [152]. HR proteins form supersized structures containing hundreds to thousands of proteins. Rad51*^Hs^* may bind B-DNA formed by trinucleotide repeats in a single strand 3′ tail. These triplets could then form Watson-Crick interactions with complementary dsDNA present in sister chromatids. After ATP hydrolysis, Rad51*^Hs^* releases a stabilized triplex DNA. By competing with Rad51*^Hs^*, RPA*^Hs^* (Replication Protein A) binds to single-stranded DNA with high affinity providing protection against nucleases [153]. Strand invasion can lead to crossover resolution and or to non-crossover resolution (patch) sequence exchange at Holiday junctions [154].

#### 3.5.2. HR in Yeast

As in mammals, HR is initiated by resection, which is a two steps process. Initially, a 50–200 nt resection is performed by the MRX*^Sc^* (Mre11*^Sc^*/Rad50*^Sc^*/Xrs1*^Sc^*) complex and Sae2*^Sc^* in *S. cerevisiae*, and MRN*^Sp^* (Mre11/Rad50/Nbs1*^Sp^*) complex and Ctp1*^Sp^* in *Sc. pombe*, which are respectively MRN*^Hs^* and CtIP*^Hs^* homologs [155,156]. Then, resection extends to kilobases through the action of Exo1*^Sc^* or Sgs1*^Sc^*-Top3*^Sc^*-Rmi1*^Sc^* (STR*^Sc^*) complex with Dna2*^Sc^* endonuclease in *S. cerevisiae* [157]. Exo1*^Sp^* is likely the HR exonuclease in *Sc. pombe*, but Exo1*^Sp^* deletion does not increase sensitivity to ionizing radiation, suggesting functional redundancy. Exo1*^Sp^*-Rad50*^Sp^* double mutant displays enhanced sensitivity than individual mutant, showing distinct activities [156]. After resection, single-strand tails are coated by RPA homologs (RPA*^Sc^* in *S. cerevisiae* and Rad11*^Sp^* in *Sc. pombe*) in order to protect them and prevent untimely Rad51*^Hs^* homologs (Rad51*^Sc^* in *S. cerevisiae* and Rhp51*^Sp^* in *Sc. pombe*) binding [158]. In *Sc. pombe*, deletion of the Rad11*^Sp^* gene is lethal, and the temperature-sensitive mutant (Rad11-404) shows high sensitivity to genotoxins [159]. In *S. cerevisiae*, Rad52*^Sc^* displaces some RPA*^Sc^* molecules to allow Rad51*^Sc^* binding to ssDNA and to initiate the nucleoprotein filament formation [160]. Rad52*^Sc^* can be assisted by Rad51*^Sc^* paralogues, including Rad55*^Sc^*-Rad57*^Sc^*, which has been shown to stabilize Rad51*^Sc^* filaments [161]. The ssDNA invasion by Rhp51*^Sp^* nucleofilaments, in *Sc. pombe*, results from a competition between Rhp51*^Sp^* and Rad11*^Sp^* for ssDNA binding. Deletion of the Rhp51*^Sp^* gene leads to much higher sensitivity to ionizing radiations [162]. Rph51*^Sp^* binds to ssDNA to form the nucleoprotein filaments in an ATP-dependent manner [163]. As in mammals, the Rad51*^Sc^*/Rhp51*^Sp^* filament promotes strand invasion to find a DNA duplex with sufficient homology to form a synapse. Although very complex, the process can take from 20 to 60 min to complete [164,165]. In *Sc. pombe*, HR is involved in processing stalled replication forks [166]. Not surprisingly, mutations of HR proteins lead to alteration of DNA replication. Moreover, Segurado et al. [167] have shown that DNA replication is associated with physical interactions and annealing between sister chromatids. Such structures are not found in Rad22*^Sp^* (Rad52*^Hs^* homolog), or Rhp51*^Sp^* mutants, clearly linking HR to replication. Finally, Rhp51*^Sp^* and Rad22*^Sp^* foci induced by fork stalling, result in chromosomal rearrangements from an increase in recombination activity [168].

### 3.6. Non-Homologous End-Joining

#### 3.6.1. General Mechanism

In mammals, Non-Homologous End-Joining (NHEJ) is a DSBs repair pathway that allows direct ligation of DNA ends in an error-prone manner (Figure 3). NHEJ proceeds in three tightly regulated steps. First Ku70/80*^Hs^* heterodimers bind to free DNA ends and act as protein hubs [169]. DNA-Pkc*^Hs^* (DNA dependant protein kinase catalytic subunit) is recruited given its high affinity for the Ku-DNA complex [170]. DNA-Pkc*^Hs^* undergo an auto-phosphorylation that recruits and activates other repair partners [171]. The second step consists of DNA end processing by the Artemis*^Hs^* endonuclease [172]. A third step involves XRCC4*^Hs^* and XLF*^Hs^* (XRCC4-Like factor or Cernunnos*^Hs^*), which interact together to form a small filament [173]. This allows interaction between the two DNA ends and the action of DNA ligase IV*^Hs^*. These canonical NHEJ proteins are sufficient to repair some DSBs, but a number of DSBs require more proteins including nucleases, DNA polymerases, and some functional homologs of XRCC4*^Hs^* and XLF*^Hs^* [174].

#### 3.6.2. NHEJ in Yeast

NHEJ Ku70*^Hs^*, Ku80*^Hs^*, and lig IV*^Hs^* homologs are present in both *S. cerevisiae* (Yku70/80*^Sc^*, Dnl4*^Sc^*) and *Sc. pombe* (Pku70/80*^Sp^*, Lig4*^Sp^*), but DNA-PKcs*^Hs^* and Artemis*^Hs^* homologs proteins have yet to be identified. Whereas *Sc. pombe* only possesses an XRCC4*^Hs^* homolog, Xlf1*^Sp^*, *S. cerevisiae* possesses both XRCC4*^Hs^* (Nej1*^Sc^*) and XLF*^Hs^* (Lif1*^Sc^*) homologs [175,176,177,178,179,180]. In yeasts, MRX is also related to NHEJ, as recently shown in *Sc. pombe,* where it seems to play a similar role as Artemis*^Hs^* and processes dirty ends before ligation. This function is enhanced by Sae2*^Sc^*/Ctp1*^Sp^* (CtIP*^Hs^* homologs) [178,181,182].

### 3.7. Single-Strand Annealing

#### 3.7.1. General Mechanism

Single Stranded Annealing (SSA) arises when a DSB is formed between two homologous regions. Once the repair is completed, the sequence between repeats is lost. Repeats must be separated by at least 400 bp and up to 15 kbp [183]. The first step in SSA depends on CtIP*^Hs^* and Mre11*^Hs^* resection (Figure 3). This resection is around 100 nt in length and could be targeted by MMEJ [184]. Once generated, a 3′ single-strand tail is covered by RPA*^Hs^* and Rad51*^Hs^* [185]. RPA*^Hs^* is progressively displaced to allow Rad51*^Hs^* filaments formation. The scanning for strand homology is provided by the activity of Rad52*^Hs^*. After annealing, the two 3′ tails emerging from the hybridized region are targeted by ERCC1*^Hs^*-XPF*^Hs^* for cleavage with the help of Rad52*^Hs^* [186]. Gaps between newly hybridized and the original dsDNA are then filled by a combination of DNA polymerase and ligase [133].

#### 3.7.2. SSA in Yeast

*S. cerevisiae* and *Sc. pombe* SSA involves a similar sequence of events: DNA resection, annealing, 3′ overhang tail removal by Rad1*^Sc^*-Rad10*^Sc^*/Rad16*^Sp^*-Swi10*^Sp^* flap endonucleases (Figure 3). *Sc. pombe* SSA is functionally linked to Rad16*^Sp^* and Swi10*^Sp^* [187], as well as Rad22*^Sp^* but not to Rhp51*^Sp^*, suggesting a specific SSA pathway in this model. However, in *Sc. pombe*, mutation of Rad16*^Sp^*-Swi10*^Sp^* results in a very small increase in sensitivity to ionizing radiations [188,189], suggesting that SSA is not a major repair pathway in this experimental model.

### 3.8. Yeast Specific DNA Repair Mechanisms

#### 3.8.1. UV Damage Excision Repair

UV-induced damages can also be resolved by an alternative pathway discovered in yeast *Sc. pombe* [190]. Since NER genes deletion, mutants of either Rad16-Swi10*^Sp^*, Rad13*^Sp^*, Rad15*^Sp^*, or Rad16*^Sp^* show unaltered sensitivity to UV, an alternative system has been proposed and termed “UV Damage Excision Repair” (UVDER) that relies on the UV Damage Endonuclease, UVDE. Uvde*^Sp^* enzyme can perform 5′ incision just next to CPD, 6-4 photoproducts, or to Dewar isomers [191]. In contrast to photolyases or glycosylases, Uvde*^Sp^* recognises both CPD and 6–4 photoproducts and may process other substrates, as it is compatible with a nuclease activity reported over apurinic/apyrimidinic sites. However, the deletion of UVDE shows a weak increase in sensitivity to chemical genotoxins [192,193]. In *Sc. pombe,* the Rad2*^Sp^* protein, a homolog of mammalian FEN1*^Hs^* or *S. cerevisiae* Rad27*^Sc^*, could be involved in a related NER alternative pathway. Indeed, Rad2*^Sp^* mutants are far more sensitive to UV than Uvde*^Sp^* mutants whereas Uvde-Rad2*^Sp^* double mutants are less sensitive than single Rad2*^Sp^* mutants [194]. This is compatible with a mechanism whereby Rad2*^Sp^* could help to process Uvde*^Sp^* nicked DNA, and where the lack of Rad2*^Sp^* leads to a potentially lethal accumulation of Uvde*^Sp^*-lesions [195]. Whatever the activation mechanism, UVDER-induced lesions can be repaired by HR through the activity of both Rhp51*^Sp^* and Rad18*^Sp^* [196]. Moreover, it has been shown that Exo1*^Sp^* endonuclease may also process Uvde*^Sp^* nicked intermediates, as Exo1*^Sp^* and Exo1-Uvde*^Sp^* mutants have similar phenotypes [197].

#### 3.8.2. Photolyases

In contrast to mammals and fission yeast, budding yeast possesses two unique DNA repair enzymes: Phr1*^Sc^* and Mgt1*^Sc^*. Phr1*^Sc^* is a DNA photolyase, or a so-called CPD-specific photolyase [198,199]. It is produced in response to UV-C, or to exposure to alkylating agents [200]. Phr1*^Sc^* is a monomeric protein with two prosthetic residues and uses near-UV photon (300–500 nm) to split off the cyclobutane ring of CPD, repairing DNA [201]. The second unique enzyme, Mgt1*^Sc^*, is a methyltransferase, targeting O6-MeG and O4-MeT [202]. O6-MeG can mismatch with thymine whereas O4-MeT mismatches with a guanidine, leading to G:C->A:T and to A:T->G:C transversions. In a suicide reaction, Mgt1*^Sc^* transfers the faulty methyl to its cysteine, leading to its inactivation [202,203]. Despite its repair abilities, Mgt1*^Sc^* has a higher affinity for O6-MeG than O4-MeT, resulting in a bias in repair kinetics.

### 3.9. Alternative DNA Repair Approaches in Yeast

Despite their evolutionary distance, NER repair systems are relatively similar between the two yeast models. However, in *Sc. pombe*, the presence of UVDER conceals certain phenotypes of NER proteins mutants, suggesting a strong functional redundancy. However, this is nuanced in the presence of UV, because of *S. cerevisiae* Phr1*^Sc^* photolyase which processes NER substrates and somewhat hides NER deficient phenotypes. The TC-NER sub-pathway is also favoured in *S. cerevisiae* by the Rpb9*^Sc^* subunit of RNA polymerase, allowing rapid identification of RNA pol II arrests leading to redundancy in TC-NER initiation. On the other hand, *Sc. pombe* has two homologs of XPC*^Sp^* (Rhp41*^Sp^* and Rhp42*^Sp^*). Although their targets are slightly different, this redundancy seems to indicate the greater importance of GG-NER.

The case of the BER system is more difficult to compare. Although the general process is similar, specificity in glycosylases creates subtle differences in processes. Thus, even if *Sc. pombe* has two UDG, against one in *S. cerevisiae*, it does not have Ogg1*^Sc^*. Oddly enough, Ogg1*^Hs^* is in charge of the most common damage, 8-oxoG, in mammals, but this does not appear to be the case in budding yeast. This useful function is performed by Ntg1*^Sc^*/Ntg2*^Sc^* and by Nth1*^Sp^*, respectively in *S. cerevisiae* and *Sc. pombe*. The redundant Ntg*^Sc^* homologs in budding yeast are required because of their different subcellular localization and not to improve repair fidelity. Bifunctional glycosylases seem to be more efficient for cells as it reduces the number of steps in the BER process. Thus, *S. cerevisiae* has 3 bifunctional enzymes, compared to only one in fission yeast. However, Nth1*^Sp^*, the bifunctional enzyme in *Sc. pombe,* is a key component of BER, apparently acting upstream of AP endonucleases. Regarding alkylation, although *Sc. pombe* has two glycosylases dedicated to this task, Mag1*^Sc^* from *S. cerevisiae* seems much more efficient. Perhaps this is due to the prominent role of NER and HR in the repair of alkylation in fission yeast. However, in addition to having a more efficient enzyme, *S. cerevisiae* has a photolyase, Mgt1*^Sc^*, specific to this damage, and provides a more efficient BER activity in this model. *Sc. pombe* has one more enzyme, Myh1*^Sp^*, which is in charge of resolving mismatches in front of an adenine. This may be related to a simpler MMR mechanism in this model. Few differences seem to emerge regarding AP endonucleases, although abasic sites may require processing from activation of Uve1*^Sp^*, in the *Sc. pombe* UVDER system to complete BER more efficiently.

As outlined previously, the MMR system in fission yeast seems much simpler considering that MutSβ*^Sp^* has lost its function in MMR and that MutLα*^Sp^* is the only homolog of MutL. This observation was also made in other species, such as *Drosophila* and *Caenorhabditis*, suggesting a lesser role for MMR in maintaining genomic integrity. On the other hand, *Sc. pombe* can rely on alternative pathways, for example using NER machinery. However, this is compensated for by the existence of short patches repair in *S. cerevisiae*, although it does not involve the machinery of NER.

The two yeasts respond to UV damage in different ways. Response to UV in *S. cerevisiae* is elegant because it uses the energy of the toxic UV, to repair the damage with Phr1*^Sc^* photolyase. This however is too specific to CPDs, as UV can induce other damage such as 6–4 photoproducts and Dewar isomers. *Sc. pombe* adapted by using the UVDER system which can recognize all these different substrates. UVDER is so efficient that it compensates for a defective NER and can support BER in the elimination of abasic sites.

The HR system is predominant in both yeasts and appears to work in similar ways. However, the STR*^Sc^* complex confers a greater resection activity to *S. cerevisiae*. The filament also appears to be better stabilized by Rad51*^Sc^* homologs. In *Sc. pombe,* stalls of the replication fork are repaired by HR, so it ensures proper replication in this model.

The NHEJ systems are seemingly different between these two models. In *S. cerevisiae,* the MRX*^Sc^* complex seems very important to NHEJ. Since MRX*^Sc^* is known to direct resection, its role in NHEJ is rather unexpected. In *S. cerevisiae*, MRX*^Sc^* is used mainly to process dirty DNA ends, as Artemis*^Hs^* does in mammals. This suggests that resection is important in *S. cerevisiae* for repairing DSBs regardless of the repair mechanism. Recently, MRN*^Sp^* has been shown to influence the NHEJ system in *Sc. pombe*, albeit with limited action, as shown by mutants of Rad50*^Sp^*, which do not change NHEJ repair capacity. It is likely that the lesser dependence on MRN*^Sp^* in *Sc. pombe* protects against extensive resection which, in a haploid cell, could be dangerous.

SSA systems of the two yeast models appear to also be different but have been studied to a much lesser extent. In fact, in *Sc. pombe* SSA was shown not to rely on Rhp51*^Sp^*, the core protein of HR. However, since SSA does not depend on the presence of sister chromatids, its regulation is somehow opposite to the activities of HR proteins. An HR-independent pathway, like SSA, should therefore be more valuable in the haploid fission yeast. Unfortunately, as the activation of SSA is related to the presence of MRN*^Sp^*, but this protein is mostly expressed in the presence of sister chromatid, the moment where SSA competes with HR, SSA is relegated to a secondary system in fission yeast.

## 4. Repair in Human Germ Cells

### 4.1. Gametes

Most vertebrates are anisogametic whereby gametes from the opposite sex are specialized and differ in morphology and number. They are therefore expected to respond differently to DNA damage inducers. This is especially the case for mammalian gametes where spermatozoa are ex-soma cells produced in large numbers whereas the oocyte is a resident cell. One additional major difference is the much greater level of DNA compaction provided by protamines in mature sperm following the eviction of most histones whereas oocytes maintain histone-based chromatin.

In addition to the greater number of cell divisions required to produce mature sperm, the peculiar steps of the sperm differentiation program may be more vulnerable to both exogenous and endogenous DNA damage inducers. For instance, endogenous DNA breaks may arise from the change in chromatin structure in post-meiotic spermatids coincident with a decreased DNA repair activity of these cells, whereas oocytes possess a far greater DNA repair capacity and harbour much fewer DNA breaks.

#### 4.1.1. Spermatozoa

Male gametogenesis is a complex differentiation program producing mature spermatozoa from spermatogonia. During meiosis, HR takes place during the late phase of prophase I. At the beginning of prophase I, programmed DSBs allow parental alleles to recombine [204]. After meiosis, spermiogenesis undergoes a Golgi phase whereby spermatozoa head and axoneme are formed. Then chromatin is remodelled with Transition Proteins (TP1 and TP2) followed by protamination where histones are replaced by protamines (PRM1 and PRM2) to achieve the highest level of DNA compaction known to the eukaryotic world.

In spermatozoa, DNA damages, strand breakage, or base alteration may result from (1) Faulty compaction/protamination of DNA [205]; (2) Abortive apoptosis, or “anastasis”, in late spermiogenesis [206]; (3) Oxidative stress [207]; (4) Persistence of enzymatic DNA breaks induce during chromatin remodelling. The contribution of each to sperm DNA damages is still unknown but it becomes clear that an elevated number of DNA breaks is associated with male infertility [208] or with impaired development of the early embryo. Chromatin remodelling in spermatids leads to the replacement of 90–95% of histones by protamines [209]. During the eviction of histones, it is assumed that free DNA supercoils are formed that can be eliminated by the action of topoisomerases [210]. Potential hindrance of topoisomerases catalytic cycle in this context may lead to SSBs in the case of topoisomerase I, or DSBs in the case of a topoisomerase II activity. A possibility also exists that mechanical breaks resulting from the major chromatin remodelling can also be created. Protamination itself can induce damage [211,212] as the PRM1/PRM2 ratio can impact proper DNA packaging [209,211,213]. We, however, generated evidence that DSBs were indeed generated during this process in the whole cell population and harbour a 3′ OH so is consistent with endonuclease digestion. In this haploid context, only direct end ligation repair mechanisms such as NHEJ are expected [214]. Although apoptosis was shown to maintain the germ cell vs Sertoli cell ratio [215,216], canonical apoptosis may not be responsible for the transient DNA breaks shown in spermatids as these are observed in 100% of spermatids. This could represent an “apoptosis-like” reversible mechanism that is reminiscent of “anastasis”, or the recovery from apoptosis described recently [217]. Various degrees of recovery, or repairs, would then modulate the persistence of DNA breaks in the mature sperm. At other spermatogenesis steps, DSBs could be linked to the altered balance of anti and pro-apoptotic factors through different steps of spermatogenesis [218] and to the compartmentalisation of the mitochondria which prevents nucleus-organelles exchanges. Reactive oxygen species (ROS) are a common source of DNA damage. ROS create abasic sites, base modifications, inter-strand crosslinking, and both single and double-strand breaks. However, ROS are essential for some sperm functions including capacitation or acrosome formation and are produced in the sperm mitochondria [219,220]. Sperm and white blood cells are the main sources of ROS in semen. Sperm cells are very sensitive to ROS because of their high content in unsaturated fatty acids and their weak DNA repair activity [207,219]. Antioxidants appear to balance a high level of ROS [221], for this purpose, seminal fluid possesses catalases, superoxide dismutases, and glutathione peroxidases and is rich in vitamins C, E, A, lactoferrin, and Q10. Ultimately, prostasomes can decrease the release of ROS from leukocytes [222]. The relevance of ROS in male infertility is emphasized as 20 to 88% of sub-fertile men show an elevated level of ROS [223].

So far, evidence of NER, BER, SSA, MMR, HR, and NHEJ DNA repair processes has been reported during spermatogenesis.

Important variations in NER activity were reported throughout spermatogenesis. For instance, spermatogonia were shown to be more sensitive to UV than meiotic or post-meiotic cells [224]. 6–4 photoproducts repair activity is also reduced significantly in post-meiotic round spermatids and during aging [224]. This may be linked to a decrease in NER protein expression [225]. Various levels of GG- or TC-NER also exist as 16.8% of CPD are removed from transcribed Scp1 gene within 16 h in rat spermatocytes [226], while 50% of CPD are removed from Dhfr and Dazl transcribed genes in spermatogonia [224]. However, spermatogonia can repair both active and inactive genes on both strands, in contrast to meiotic cells where transcribed genes are repaired. TC-NER is lower in round spermatids than in spermatogonia. These differences may reflect the greater mitotic activity of spermatogonia, and the fact that they are lying outside of the blood-testis barrier and so are potentially more exposed to genotoxins. Chromatin compaction during spermiogenesis may also adversely impact NER [227] as chromatin access is known to hinder NER. NER is therefore present at all stages of the male germ cells differentiation albeit with various efficiency.

OGG1*^Hs^* glycosylase is the only BER factor found in spermatozoa and recognizes 8-oxoG [219]. However, as none of the AP endonucleases exist in spermatozoa, AP sites will likely be processed during the first zygotic mitosis [219].

HR is known for its primary role during meiosis but is also important for DSBs and inter-strand lesions repair in spermatogonia. Outside of meiosis, HR acts in the S and G2 phases of the cell cycle, when sister chromatids are present. HR is obviously absent in haploid spermatids but resumes in the diploid zygote.

In male germ cells, some canonical components of NHEJ are missing resulting in an alternative NHEJ pathway [228]. For instance, Ku70*^Hs^* and 53BP1*^Hs^* are not expressed therefore preventing the DNA-PKc*^Hs^* pathway. PARP-1*^Hs^* and XRCC1*^Hs^* are however expressed in elongating spermatids supporting alt-NHEJ pathway as described by Ahmed et al. [228]. PARP-1*^Hs^* may bind to DSB as a catalytic homodimer bringing together both DNA ends. Then, PARP-1*^Hs^* may recruit the XRCC1*^Hsc^*/DNA ligase III*^Hs^* complex at the DSB site to complete the end-joining.

#### 4.1.2. Oocyte

Since a limited number of oocytes can be retrieved, the DNA damage response in this gamete is studied to a lesser extent. DNA damages arise mainly during the active life of oocytes in prenatal stages, before prophase I arrest, and during recovery from pre-ovulatory meiosis [229].

From the primary oocyte stage to meiosis II and ovulation, oocytes can actively repair DNA [230]. During oogenesis, repair genes are overexpressed, and their mRNA are accumulated to be used in the zygote [231]. NER, BER, MMR, HR or NHEJ repair pathways have been described in oocytes from humans to mice [231,232,233,234]. Proper DNA repair machinery must operate as oocytes undergo chromatin remodelling during fertilisation and early steps of a zygote. Oocytes are diploid cells, that possess homologous chromosomes until meiosis II that arises after fertilisation. Thus, the oocyte is virtually never haploid. The oocyte becomes transiently haploid between meiosis II end and the fusion of parental genomes (syngamy). Thus, following meiosis II, only NHEJ should operate as shown in mice [235], although variations exist between species as rodent oocytes have better DNA repair abilities than primate ones [232]. The repair capacity of the oocyte and mRNA levels for repair proteins also decreases with female age [236]. After fertilisation, syngamy is a critical step since (1) Chromatids from each parent exist in different cellular compartments; (2) Chromatin undergoes major remodelling associated with demethylation; (3) Male derived chromatids are repaired by the oocyte DNA repair machinery [237].

As for spermatogenesis, ROS are produced during oogenesis. ROS production is necessary for folliculogenesis, oocyte maturation, ovulation, and luteal function [238]. Maturation and ovulation are linked to inflammatory processes, characterized by high ROS production [239]. Unlike sperm, oocytes are more resistant to ROS due to an environment rich in antioxidants in the follicular fluid [240]. However, ROS imbalance in the female tract decreases fertility and oocyte quality [241]. In addition to its impact on genetic integrity, oxidative stress is known to alter microtubule function and spindle morphology leading to aneuploidy [242].

In contrast to spermatozoa, OGG1*^Hs^* glycosylase is only weakly expressed in oocytes, as 8-oxoG damages are not present in spermatozoa after fertilization [243]. In many aspects, the early zygote may still be considered as an oocyte since only mRNAs for the oocyte are used up until the 4-cell stage [244]. This “zygotic oocyte” is in charge of repairing DNA damages in spermatozoa but may be overloaded if DNA damages occur in more than 8% of the nucleotide genome amount [245,246].

Both the number and the nature of DNA breaks are of concern for fertility. Whereas SSBs are easily repaired as in somatic cells, DSBs can overload the DNA repair capacity of the oocyte. After fertilisation, the persistence of DSBs in spermatozoa will delay DNA replication or can even be lethal for the zygote [247]. Finally, DSBs are mainly repaired by NHEJ during zygote [214], as for somatic cells.

## 5. Conclusions

Ploidy is a key element to consider in the regulation of DNA repair systems. In view of the aforementioned details about the two yeast models, it seems that species can however adapt their uses of DNA repair systems to overcome the weaknesses of either haploidy or diploidy. Thus, many redundancies exist within and between DNA repair systems that are linked together into a complex and integrated overarching regulatory mechanism that ensures the maintenance of genomic integrity regardless of the ploidy state. This may of course represent a functional bias for a single-cell organism as they are solely responsible to generate genetically intact progeny. In fact, major differences in repair efficiency between diploid and haploid cells are mainly observed among mammalian metazoan gametes. Hence, unlike oocytes, mature sperm clearly lacks proper DNA repair activity. However, the many replication/differentiation steps, change in DNA topology and highly condensed and specialized chromatin structure observed during spermatogenesis offers an appropriate context to generate adaptative variations.

## Figures and Tables

**Table 1:** Table of homologous proteins.

	*H. sapiens*	*S. cerevisiae*	*S. pombe*	Functions
TC NER	RNA pol II	RNA pol II	RNA pol II	mRNA synthesis
	CSB	Rad 26	Rhp 26	Detection of RNA pol II arrest
	-	Rpd 9	-	Part of RNA pol II, detection of arrest
	CSA	Rad 28	Ckn1	TC-NER protein hub
	UVSSA-USP7	-	-	Ubiquitine ligase
GG NER	XPC	Rad4	rhp41, rhp42	Scan to detect damages
	Rad23b	Rad23	Rhp 23	XPC partner
	TFIIH	TFIIH	TFIIH	Transcription factor, check for damage
	XPB	Rad 25	Ptr 8	Core units of TFIIH (helicases)
	XPD	Rad 3	Rad 15	
	XPA	Rad 14	Rph 14	TFIIH helicases stimulator
	ERCCI-XPF	Rad 10	Swi 10	Endonuclease complex, cleaves 5′ to damage
	XPF	Rad 1	Rad 16	
	XPG	Rad 2	Rad 13	Endonuclease, cleaves 3′ to damage
BER	UNG	Ung 1	Ung 1	Uracil DNA Glycosylase (UDG)
	TDG	-	Thp 1	UDG, T:G, U:G mismatches, 5-fluorouracil, 3,N4-ethanolcytosine, 5-hydroxyuracil, Xanthine, Oxanine, Hypoxanthine DNA glycosylase
	-	Mag 1	Mag 1	Alkylation DNA Glycosylase
	-	Mag 2	Mag 2	
	OGG 1	Ogg 1	-	8-oxoG, fapy-G, 7,8-dihydro8oxoG DNA Glycosylase
	NTHL 1	Ntg1/Ntg2	Nth 1	8-oxoG, 8-hydroxycytosine, thimidineglycol, 8-hydroxyuracil DNA Glycosylase, AP site β lyase
	MUTYH	-	Myh 1	Glycosylase of adenine mismatches
	APE1	Apn 2	Apn 2	AP endonucleases
	-	Apn 1	Apn 1	
	FEN 1	Rad 27	Rad 2	Structure specific endonuclease, cleaves at ssDNA-dsDNA transition
MMR	MutS α	MutS α	MutS α	Mismatch detection
	MutS β	MutS β	MutS β	Long insertion deletion loops detection, involved in recombination
	MutS γ	MutS γ	MutS γ	Holliday Junction resolvase
	MutL α	MutL α	MutL α	MutSα and MutSβ interactor, weakly endonuclease
	MutL β	MutL β	-	
	MutL γ	MutL γ	-	MutSγ interactor
	EXO 1	EXO 1	EXO 1	Exonuclease
	PCNA	PCNA	PCNA	DNA polymerase helicase
UVDER	-	-	UVE 1	UV damage endonucleases
	-	-	UVDE	
Photolyase	-	Phr1	-	CPD specific photolyase
	-	Mgt1	-	O6-MeG, O4-MeT methyl transferase
HR	Mre11/Rad50/Nbs1	Mre11/Rad50/Xrs1	Mre11/Rad50/Nbs1	MRN/X complex, DNA ends detection, resection initiation
	CtIP	Sae2	Ctp 1	Endonuclease
	RPA	RPA	Rad 11	Single strand DNA binding protein
	Rad 52	Rad 52	Rad 22	Displace RPA to form Rad51 filament
	Rad51	Rad 51	Rhp 51	Globular protein forming filaments for strand invasion
NHEJ	Ku 70/80	YKu 70/80	PKu 70/80	DNA ends detection and protection
	DNA-Pkc	-	-	Protein kinase
	Artemis	-	-	Endonuclease, DNA ends processing
	XRCC4	Nej 1	-	DNA ends bridging by filaments formation
	XLF	Lif 1	Xlfl	
	DNA ligase IV	Dnl 4	Lig 4	DNA ends ligation
